# A novel ALG10/TGF-β positive regulatory loop contributes to the stemness of colorectal cancer

**DOI:** 10.18632/aging.204116

**Published:** 2022-06-09

**Authors:** Xiaotian Xu, Huideng Wang, Xinhui Li, Xiaoqun Duan, Yuhui Wang

**Affiliations:** 1 Guangxi Colleges and Universities Key Laboratory of Pharmacology, Guilin Medical University, Guilin 541004, China

**Keywords:** *ALG10*, TGFBR2, stemness, colorectal cancer, glycosylation

## Abstract

The roles of asparagine-linked glycosylation (ALG) members in tumorigenic process have been widely explored. However, their effects in colorectal cancer progression are still confusing. Here, we screened 12 ALGs’ expression through online datasets and found that *ALG10* was mostly upregulated in colorectal cancer tissues. We found that *ALG10* knockdown significantly suppressed the expression of stemness markers, ALDH activity, and sphere-formation ability. *In vivo* tumorigenic analysis indicated that *ALG10* knockdown attenuated the tumor-initiating ability and chemoresistance of colorectal cancer cells. Further mechanistic studies showed that *ALG10* knockdown suppressed the activity of TGF-β signaling by reducing TGFBR2 glycosylation, which was necessary for *ALG10*-mediated effects on colorectal cancer stemness; Conversely, TGF-β signaling activated *ALG10* gene promoter activity through Smad2’s binding to *ALG10* gene promoter and TGF-β signaling promoted the stemness of colorectal cancer cells in an *ALG10*-dependent manner. This work identified a novel *ALG10*/TGF-β positive regulatory loop responsible for colorectal cancer stemness.

## INTRODUCTION

Colorectal cancer (CRC) is the second most common cancer type in the world and the incidence rate and mortality rate are high, which seriously threatens human life and health [1]. In recent decades, with the proposal and development of the concept of cancer stem cells (CSCs), more in-depth exploration of CSCs-related signal pathways has been carried out [2]. CSCs are a small part of tumor cells with the ability of self-renewal, proliferation, and differentiation [3]. CSCs were isolated from CRC tissues for the first time in 2007, which were also the first CSCs found in gastrointestinal tumors [4]. Their specific surface markers and abnormally activated signal pathways are closely related to the occurrence, development, recurrence, metastasis, low survival rate and drug resistance of CRC. Therefore, the regulation of CSC related surface markers and signal pathways has become the key to the targeted treatment of CRC.

Glycosylation is the most important post-translational modification of known proteins, in which glycosyltransferase transfers glycosyl donors to glycosyl receptors [5]. Abnormal glycosylated glycoproteins often exist in malignant tumors, and glycosyltransferase plays an important role in tumor progression [6]. There are multiple types of protein glycosylation, among which the most common are N-glycosylation in asparagine, and O-glycosylation in serine and threonine. Asparagine-linked glycosylation (ALG-N-glycosylation) is an important process of protein co-translation and post-translational modification [7]. Under the catalysis of oligoglycosyltransferase, the oligosaccharide structure of Tetradecyl sugar group, which is synthesized in advance on the endoplasmic reticulum membrane, is transferred to the glycosylation site of new peptide chain (NXS/T, N is asparagine, X is any amino acid except proline, S/T is serine/threonine). N-linked glycan (N-glycan) is an important regulatory signal of endoplasmic reticulum quality control system (ERQC), which facilitates the correct folding of newborn peptide chains and the degradation of misfolded proteins (ERAD). In animal and plant cells, as the protein is secreted from the endoplasmic reticulum into the Golgi apparatus, the N-glycan carried by the protein will continue to be cut and processed to form complex glycogen. *ALG10* gene encodes a member of the glycosyltransferase 2 family which participates in the N-linked glycosylation of oligonuclear glycosylation of protein. The addition of oligonuclei of glucose residues is required for substrate recognition and thus effectively transmitted to the nascent glycoproteins. *ALG10* has been shown to be necessary for efficient N-glycosylation and leaf growth [8], and it has been demonstrated that *ALG10* is associated with arrhythmia [9]. However, the clinicopathological significance and functional role of *ALG10* in CRC progression are still unclear, which needs to be further discussed.

In this study, we screened and found that *ALG10* was up-regulated most significantly in CRC compared with other *ALG* family genes. This study aims to explore the relative expression of *ALG10* in CRC and corresponding normal tissues, and its relationship with clinical prognosis, and further reveals the underlying mechanism of *ALG10* regulating the stemness of CRC, which will be beneficial for understanding *ALG10* effects in tumorigenesis and development, and provide potential targets for the prognosis and treatment of CRC.

## MATERIALS AND METHODS

### Cell lines and reagents

CRC cell lines HT-29, CT-26, SW480, SW620, HT-29, HCT-116, SW1116 and normal colorectal epithelial cell line NCM460 were purchased from Keygen (Nanjing, China); Oxaliplatin resistant HCT-116-OR cells were purchased from Shanghai Meixuan Biological Technology Co., LTD. Cell lines were passaged in our laboratory. Cell line authentication was assessed using short tandem repeat (STR) DNA profiling method every 6 months. All of these cell lines were cultured in RPMI-1640 medium (Keygen) with 10% Fetal bovine serum (FBS, Biological Industries, Kibbutz Beit Haemek, Israel) at 37°C plus 5% CO_2_. Recombinant human TGF beta Receptor II (rTGFBR2) protein was purchased from Abcam (Cambridge, MA, USA) and 50 nM was used in this study.

### Real-time quantitative PCR (RT-qPCR)

Following the standard recommendations, total RNA was extracted from CRC cells using TRIzol (ET101-01, TransGen Biotech, China). Complementary DNA (cDNA) was synthesized by reverse transcription of RNA using Reverse transcription MMLV kit (Vazyme, Nanjing, China). RT-qPCR analysis was performed to calculate the relative expression levels of different genes using the 2^−ΔΔCt^ method with Hieff UNICON^®^ Universal Blue qPCR SYBR Green Master Mix (Cat # 11184ES03, YEASEN, Shanghai, China).

### Western blot

The experimental procedures for the extraction and detection of protein expression were performed following the protocols mentioned in the previous study [10]. The detailed information of antibodies were listed as follows: Nanog (Cat No. 14295-1-AP, 1:1000, Proteintech, Wuhan, China), ALG10 (Cat # ab124711, 1:3000, Abcam), Oct4 (Cat No. 11263-1-AP, 1:1000, Proteintech), Sox2 (Cat No. 11064-1-AP1:1000, Proteintech), TGFBR2 (Cat No. 66636-1-Ig, 1:1000, Proteintech), TGFBR1 (Cat # ab31013, 1:1000, Abcam), p-Smad2 (Cat # ab28088, 1:1000, Abcam), Smad2 (Cat No. 12570-1-AP, 1:1000, Proteintech), GAPDH (Cat No. 60004-1-Ig, 1:1000, Proteintech), Histone H3 (Cat No. 17168-1-AP, 1:1000, Proteintech) were used in this study. The full length uncropped original western blots used in their manuscript were shown in Supplementary Figure 1.

### Online analytic tool

The online analytic tool (TNMplot: https://tnmplot.com/analysis/) was used to analyze the expression of ALG family members (*ALG1*, *ALG2*, *ALG3*, *ALG5*, *ALG6*, *ALG8*, *ALG9*, *ALG10*, *ALG11*, *ALG12*, *ALG13*, *ALG14*) in CRC and normal tissues. The correlation between *ALG10* expression and survival of CRC patients was determined through another online analytic tool (R2: Genomics Analysis and Visualization Platform).

### Aldehyde Dehydrogenase (ALDH) activity detection

ALDH activity was measured in CRC cells with different treatments using an Aldehyde Dehydrogenase Activity Colorimetric Assay Kit (Cat # MAK082, Sigma, St. Louis, MO, USA) following the manufacturer’s recommendation.

### Analysis of sphere-formation ability

Heparin (4 μg/mL), hydrocortisone (0.48 μg/mL), and the MammoCult Proliferation Supplements (Stem Cell Technologies, Vancouver, Canada) were added to the mammosphere formation medium (Stem Cell Technologies, Vancouver, Canada). Cells were then cultured in the prepared medium at the 24-well low-adhesion plates (Corning costar, NY, USA) at a density of 3000–5000 cells/well. Spheres with size more than 50 μm were counted and taken picture under a microscope after 10 days.

### Flow cytometry analysis of CD133+ sub-population

CRC cells with different treatments were harvested and washed three times with PBS and stained with anti-CD133 (APC-conjugated, Cat # 393905, San Diego, CA, USA). The FACSCalibur flow cytometer (BD Biosciences, Franklin Lakes, NJ, USA) was used to detect CD133+ sub-population. The experimental results were analyzed through flow cytometry software.

### Tumor-initiating assay

Mice were operated and housed according to the protocols approved by the Ethics Committee of Guilin Medical University. Briefly, 200 μl cyclophosphamide (Cat # HY-17420, MedChemExpress, Monmouth Junction, NJ, USA) was injected for three consecutive days to further destroy the immune system of the nude mice. Then mice were injected at a density of 1×10^7^, 1×10^6^, and 1×10^5^ cells/tumor, respectively. Nude mice were euthanized with isoflurane after 10 days and tumors were separated. The ratio of stem cells was calculated using an ELDA: Extreme Limiting Dilution Analysis (http://bioinf.wehi.edu.au/software/elda/).

### Luciferase reporter assay

The detailed experimental protocol was performed as we described before [8]. *Smad2* and *TCF-1* promoter sequences were inserted into pGL3-vector, named as Luc-Smad and Luc-TCF-1, respectively. Luc-Smad or Luc-TCF-1 was co-transfected with Renilla luciferase vector into CRC cells using JetPRIME (Polyplus, Illkirch, France). 72 h later, cells were collected, lysed, and then subjected to detect the luciferase activity following the instructions in the Dual Luciferase Reporter Assay Kit (Vazyme, Nanjing, China).

### Lentivirus package and stable cell line construction

Cells were infected with vector or ALG10-kd (Negative control-LV3 shuttle plasmid, HANBIO, Shanghai, China) lentivirus according to manufacturer’s protocols. Then the stable-infected cells were screened using puromycin (Sigma, 2 μg/ml) for at least 2 weeks. The knockdown efficiency was confirmed by Western blot assay.

### Co-Immunoprecipitation (IP)

Briefly, cells were washed with pre-cooled PBS and lysed with the pre-cooled RIPA buffer containing phosphatase inhibitors. Cell suspension was centrifuged for 20 min at 4°C by 13000 rpm and supernatant were subjected to Co-IP assay using Protein A agarose beads (50%) (Cat # B23202, Bimake, Shanghai, China). The detailed procedure was previously mentioned [11].

### Statistical analysis

GraphPad Prism 5.0 software (GraphPad Software, San Diego, CA, USA) was used to complete statistical analysis. Unpaired Student’s *t*-test or one-way ANOVA (analysis of variance) was performed to analyze discrepancies between groups. Statistical analyses were carried out by Student’s *t* test. *P* values < 0.05 were regarded as statistically significant.

## RESULTS

### *ALG10* was highly expressed in CRC tissues

**Figure 1:**
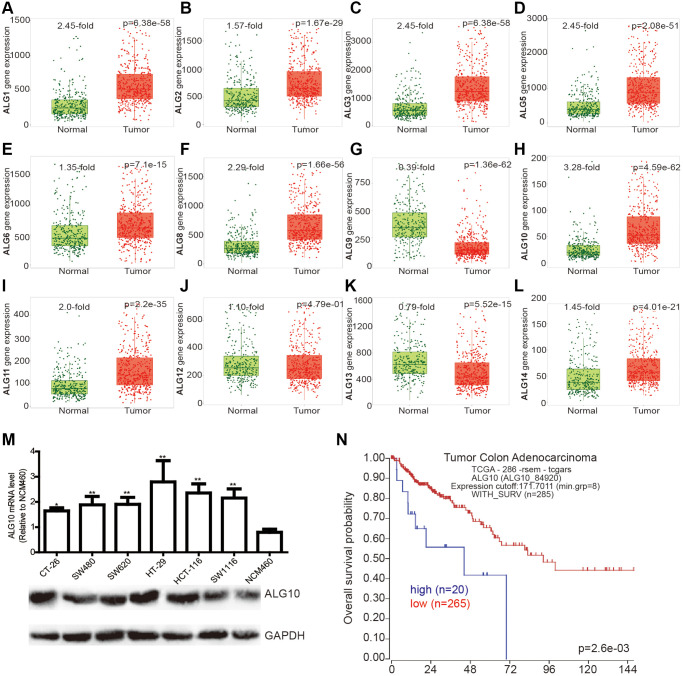
***ALG10* was highly expressed in CRC tissues.** (**A**–**L**) The expression of ALG family members (*ALG1*, *ALG2*, *ALG3*, *ALG5*, *ALG6*, *ALG8*, *ALG9*, *ALG10*, *ALG11*, *ALG12*, *ALG13*, *ALG14*) in CRC and normal tissues was evaluated through the online analytic tool (TNMplot: https://tnmplot.com/analysis/). (**M**) ALG10 mRNA and protein expression was detected in CRC cells and normal colorectal epithelial cells. *n* = 3, ^*^P < 0.05, ^**^P < 0.01 vs. Control. (**N**) The correlation between *ALG10* expression and survival of CRC patients was determined through the online analytic tool (R2: Genomics Analysis and Visualization Platform).

We initially examined the expression of ALG family members (*ALG1*, *ALG2*, *ALG3*, *ALG5*, *ALG6*, *ALG8*, *ALG9*, *ALG10*, *ALG11*, *ALG12*, *ALG13*, *ALG14*) in CRC and normal tissues through the online analytic tool (TNMplot: https://tnmplot.com/analysis/), and found that all ALG members were upregulated in CRC tissues, except for *ALG9* and *ALG13* (Figure 1A–1L). Notably, it was identified that *ALG10* was the mostly upregulated gene (3.28-fold, Figure 1H). *In vitro* cell line analysis obtained a consistent result showing that *ALG10* was highly expressed in CRC cells (Figure 1M). Additionally, the correlation between *ALG10* expression and survival of CRC patients was determined through another online analytic tool (R2: Genomics Analysis and Visualization Platform) and we found that *ALG10* expression was negatively correlated with the survival of CRC patients (Figure 1N).

### *ALG10* knockdown blocks the stemness of CRC cells

**Figure 2:**
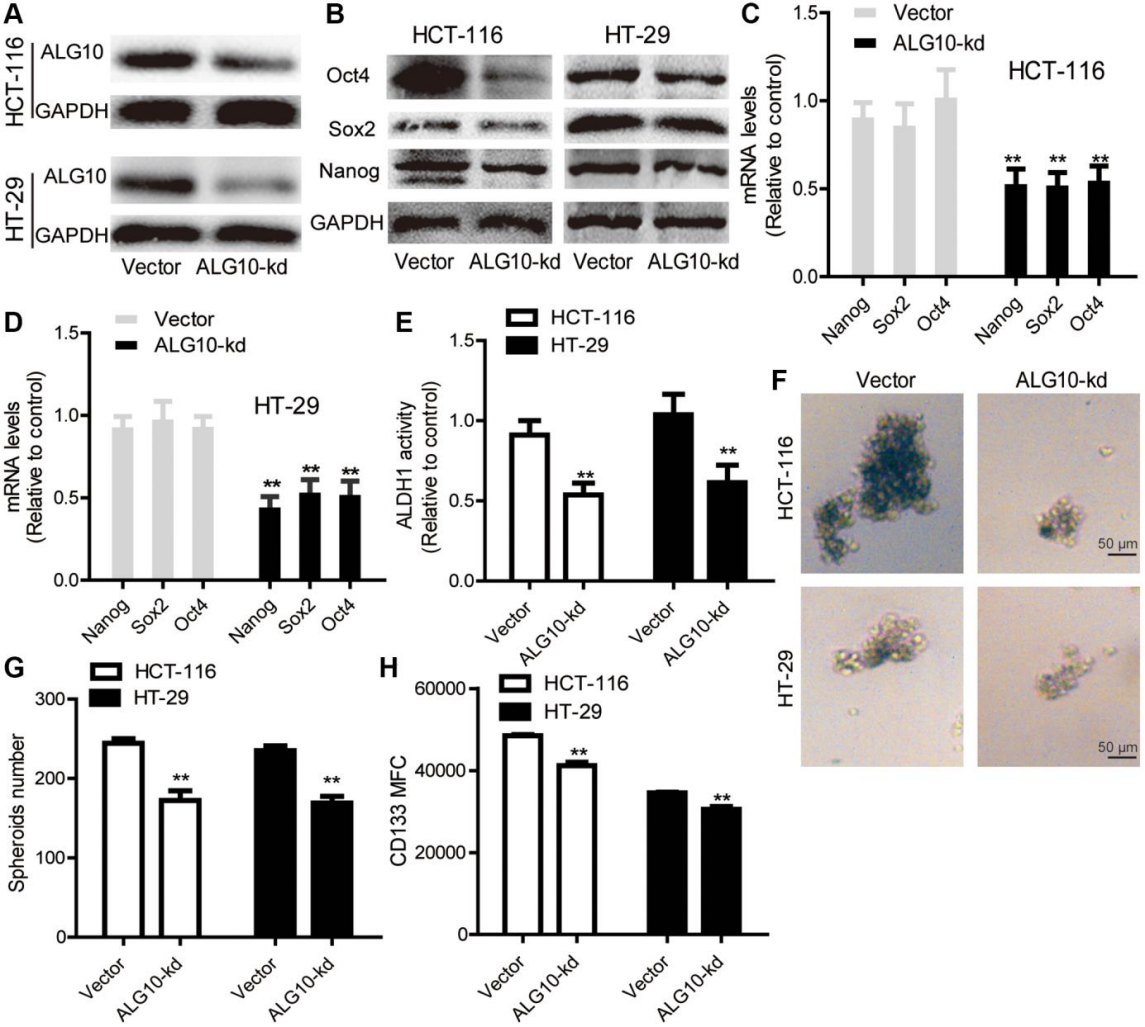
***ALG10* knockdown blocks the stemness of CRC cells.** (**A**) The knockdown efficiency of ALG10-kd was confirmed in CRC cells through western blot assay. (**B**) The protein expression of stemness markers (Oct4, Sox2, Nanog) was detected in CRC cells with or without ALG10 knockdown. (**C** and **D**) The mRNA expression of stemness markers (Oct4, Sox2, Nanog) was measured in CRC cells with or without ALG10 knockdown. (**E**) ALDH activity was determined in CRC cells with or without ALG10 knockdown. (**F** and **G**) Sphere formation ability was evaluated in CRC cells with or without ALG10 knockdown via measuring sphere size and counting sphere number. (**H**) CD133+ cell sub-population was analyzed in CRC cells with or without ALG10 knockdown. *n* = 3, ^**^P < 0.01 vs. Control.

Based on above results, we assumed that *ALG10* played an important role during CRC progression. We preferably focused on its effects on the stemness of CRC cells as CSC is regarded as the main cause of tumorigenesis. *ALG10* was knocked down in CRC cells and knockdown efficiency was confirmed by Western blot (Figure 2A). It was shown that *ALG10* knockdown inhibited the expression of stemness markers (*Nanog*, *Sox2*, *Oct4*) (Figure 2B–2D). Since ALDH activity and sphere-formation ability were remarkably upregulated in CSC [12], we further detected ALDH activity and sphere-formation ability in CRC cells with *ALG10* knockdown. As shown in Figure 2E, ALDH activity was reduced in CRC cells by *ALG10* knockdown. Moreover, the capability of sphere-formation was suppressed by *ALG10* knockdown, as characterized by the reduction of sphere number and size (Figure 2F and 2G). In agreement with these results, the CD133+ sub-population of CRC cells with stemness was attenuated by *ALG10* knockdown through flow cytometry analysis (Figure 2H).

### *ALG10* knockdown attenuates the tumor-initiating ability of CRC cells

**Figure 3:**
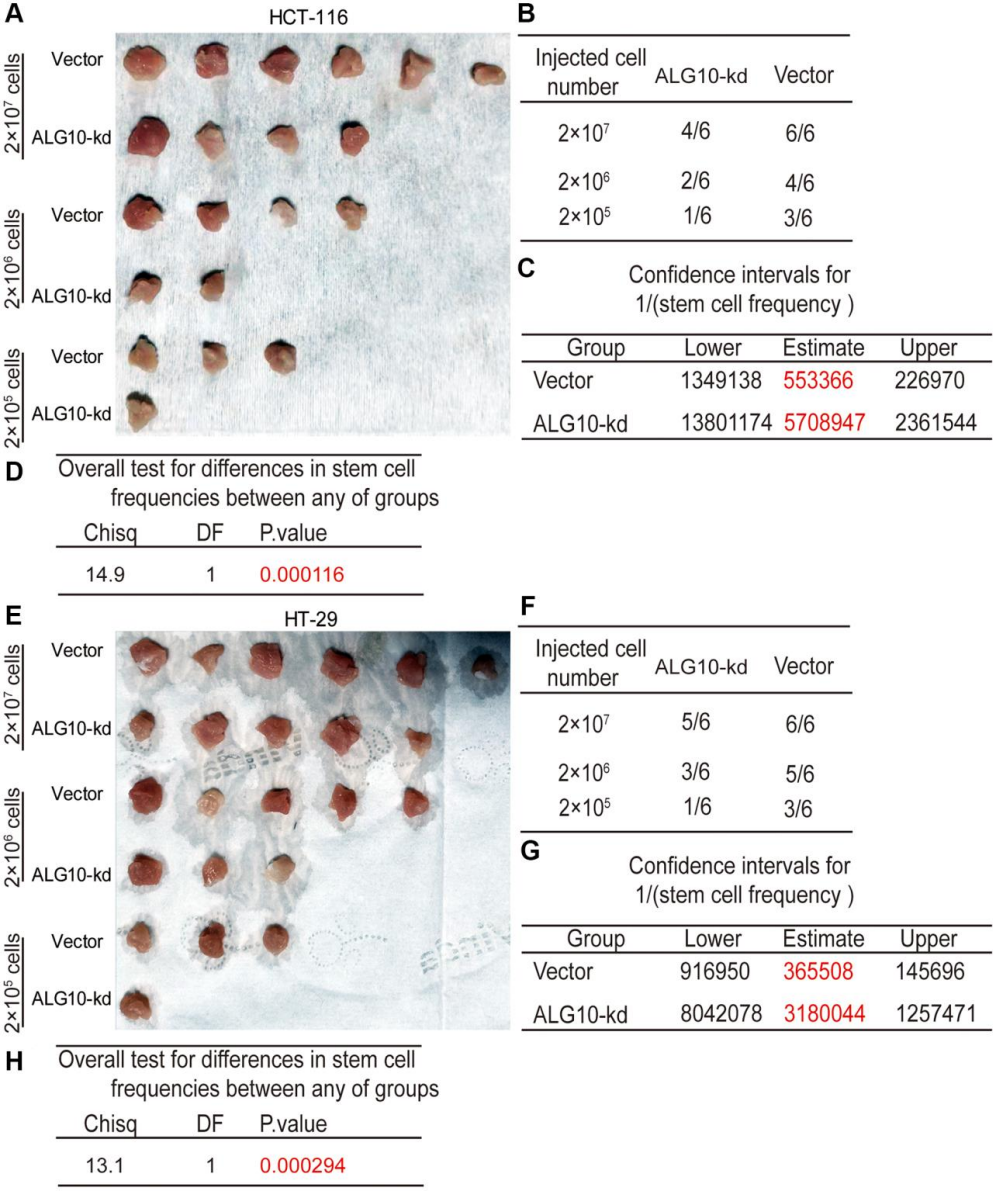
***ALG10* knockdown attenuates the tumor-initiating ability of CRC cells.** (**A**) Tumor images derived from HCT-116 cells with or without ALG10 knockdown at different cell concentrations. (**B**) Tumor formation ratio was calculated for HCT-116 cells with or without ALG10 knockdown at different cell concentrations. (**C**) The 1/(stem cell frequency) was determined for HCT-116 cells with or without ALG10 knockdown using an ELDA: Extreme Limiting Dilution Analysis (http://bioinf.wehi.edu.au/software/elda/). (**D**) The difference in stem cell frequencies between the two groups described in (**C**) was measured using an ELDA: Extreme Limiting Dilution Analysis (http://bioinf.wehi.edu.au/software/elda/). (**E**) Tumor images derived from HT-29 cells with or without ALG10 knockdown at different cell concentrations. (**F**) Tumor formation ratio was calculated for HT-29 cells with or without ALG10 knockdown at different cell concentrations. (**G**) The 1/(stem cell frequency) was determined for HT-29 cells with or without ALG10 knockdown using an ELDA: Extreme Limiting Dilution Analysis (http://bioinf.wehi.edu.au/software/elda/). (**H**) The difference in stem cell frequencies between the two groups described in (**G**) was measured using an ELDA: Extreme Limiting Dilution Analysis (http://bioinf.wehi.edu.au/software/elda/).

We tried to detect the effects of *ALG10* on the tumor-initiating ability of CRC cells, which is positively correlated the stemness of tumor cells. CRC cells with or without *ALG10*-stable knockdown were seeded into BALB/c nude mice at three different cell density as indicated. We found that the tumorigenic ability of CRC cells with *ALG10* knockdown was decreased, which was evident by the decreased tumor-formation rate (Figure 3A and 3B, Figure 3E and 3F) and stem cell frequency using the tumor-limiting dilution assay (Figure 3C and 3G). Additionally, overall test for differences in stem cell frequencies between two groups exhibited a remarkable significance (Figure 3D and 3H).

### *ALG10* knockdown attenuated the chemoresistance of CRC cells

**Figure 4:**
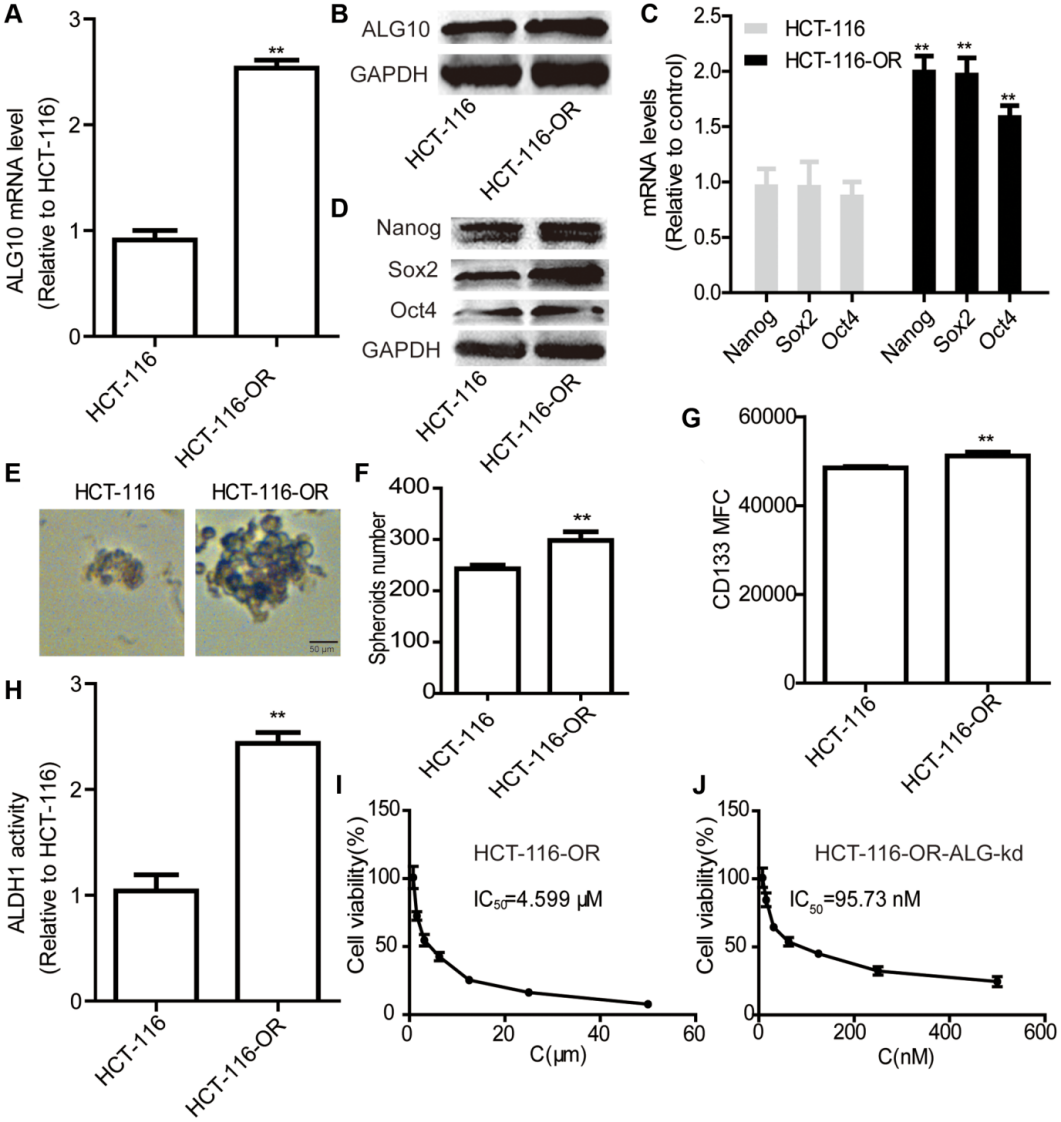
***ALG10* knockdown attenuated the chemoresistance of CRC cells *in vitro*.** (**A** and **B**) ALG10 mRNA and protein levels were examined in HCT-116 and HCT-116-OR cells. (**C** and **D**) The mRNA and protein levels of stemness markers were detected in HCT-116 and HCT-116-OR cells. (**E** and **F**) Sphere size and number were determined in HCT-116 and HCT-116-OR cells. (**G**) CD133+ subpopulation was evaluated in HCT-116 and HCT-116-OR cells. (**H**) ALDH activity was measured in HCT-116 and HCT-116-OR cells. (**I**) The IC_50_ value of oxaliplatin was calculated in HCT-116-OR cells. (**J**) The IC_50_ value of oxaliplatin was measured in HCT-116-OR-ALG-kd cells. *n* = 3, ^**^P < 0.01 vs. Control.

**Figure 5:**
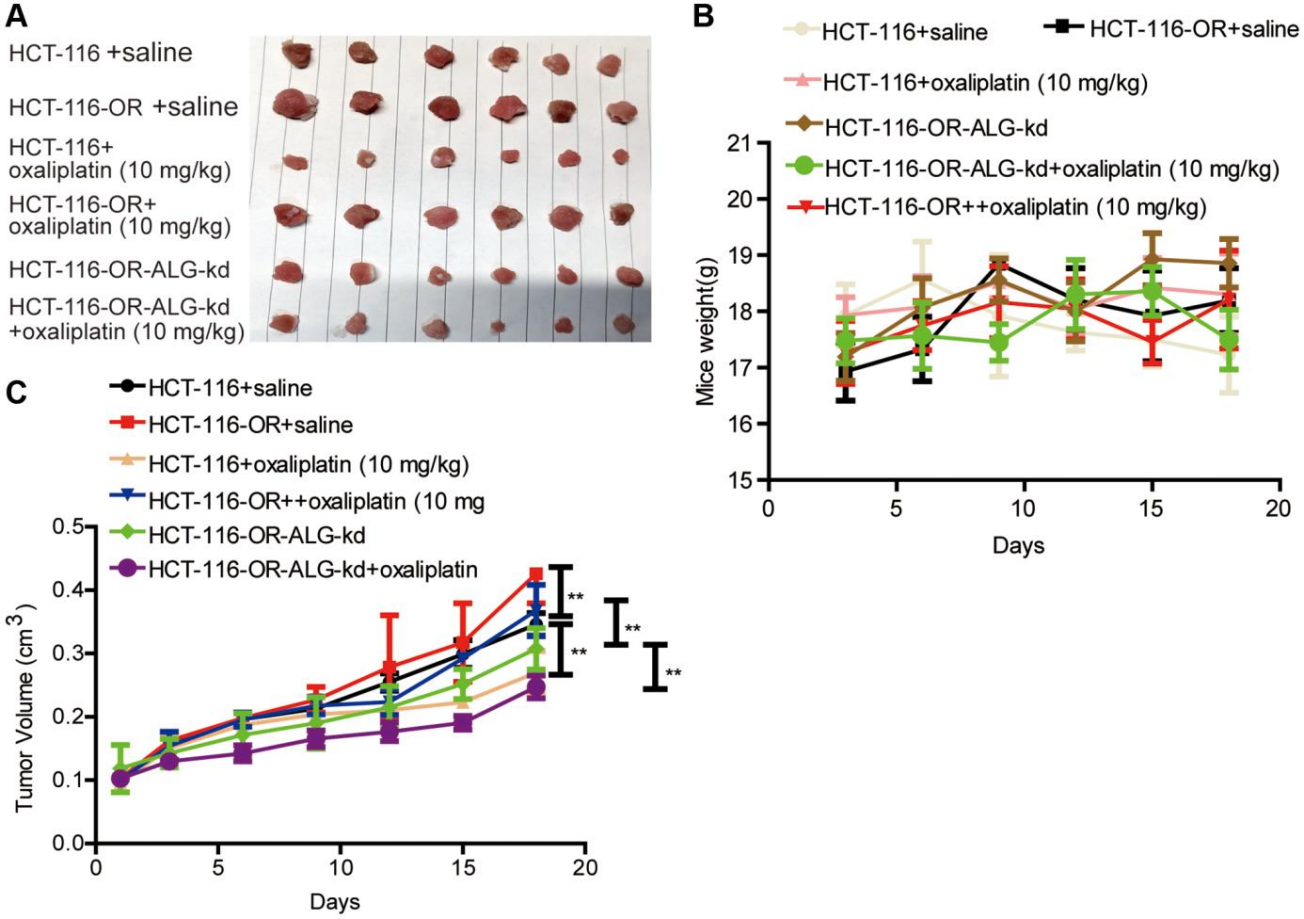
***ALG10* knockdown attenuated the chemoresistance of CRC cells *in vivo*.** (**A**) Tumor images derived from HCT-116, HCT-116-OR and HCT-116-OR-ALG10-kd cells with different treatment as indicated in figure. (**B**) Mice weight was measured at different time-points as indicated in different groups. (**C**) Tumor volume was recorded at different time-points as indicated in different groups. *n* = 6, ^**^P < 0.01 vs. Control.

Since chemoresistance has been shown to be led by the existence of CSCs, we wondered whether *ALG10* contributed to chemoresistance in CRC cells. As shown in Figure 4A and 4B, both *ALG10* mRNA and protein levels were upregulated in HCT-116-OR cells compared to that in HCT-116 cells. Indeed, HCT-116-OR exhibited a stronger stemness than HCT-116 cells, which was characterized as the increase of stemness marker expression (Figure 4C and 4D), sphere-formation ability (Figure 4E and 4F), CD133+ subpopulation (Figure 4G), and ALDH activity (Figure 4H). Additionally, it was found that *ALG10* knockdown attenuated oxaliplatin resistance of HCT-116-OR cells *in vitro*, which was evident by the decrease of IC50 value of oxaliplatin (4.599 μM vs. 95.73 nM) (Figure 4I and 4J) and *in vivo* (Figure 5). These results suggest that *ALG10* can promote the stemness and chemoresistance of CRC cells.

### *ALG10* knockdown suppressed the activity of TGF-β signaling by reducing TGFBR2 glycosylation

**Figure 6:**
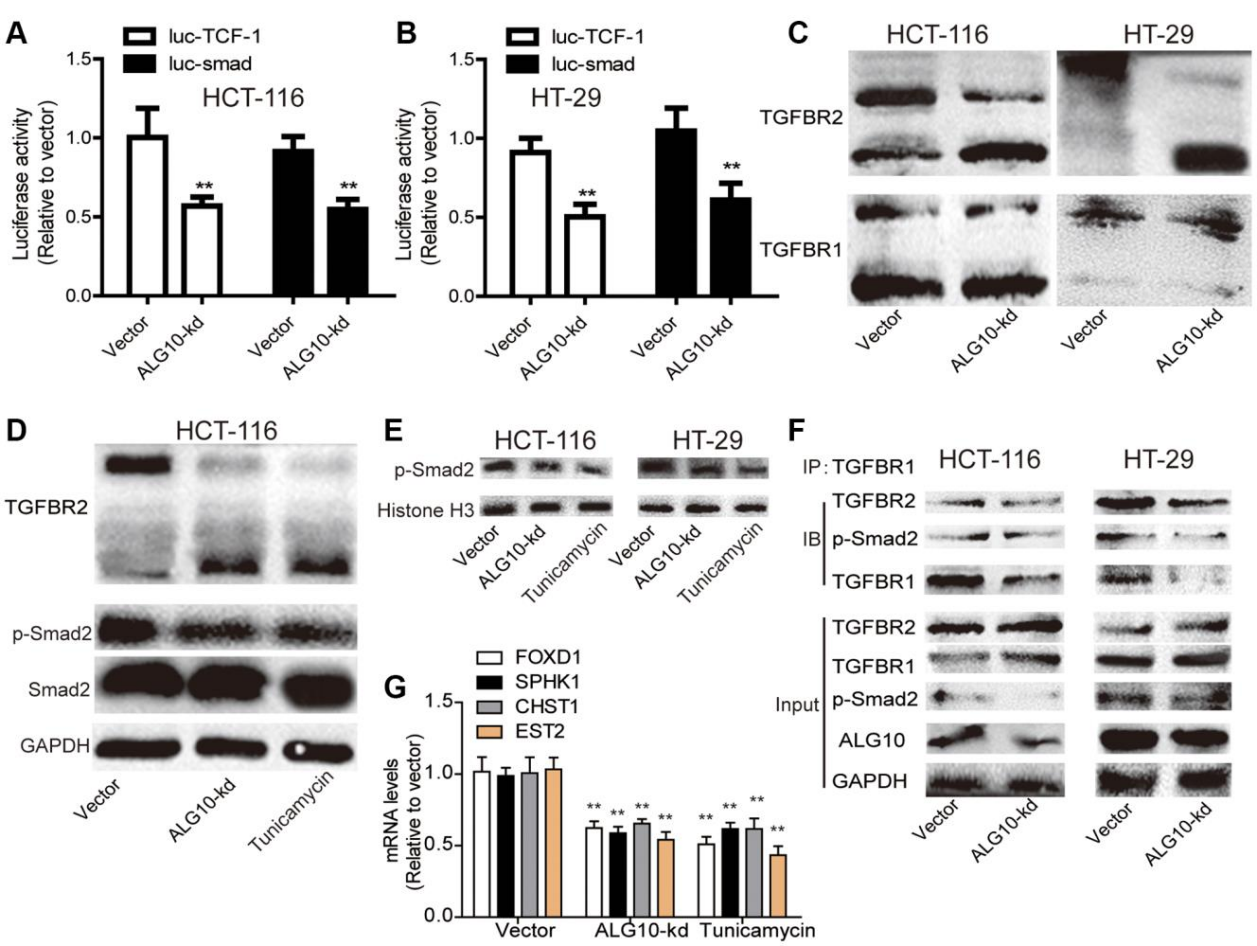
***ALG10* knockdown suppressed the activity of TGF-β signaling by reducing TGFBR2 glycosylation.** (**A** and **B**) The activity of Luc-TCF-1 and Luc-smad was evaluated in HCT-116 and HT-29 cells with or without ALG10 knockdown. (**C**) The status of TGFBR1/2 glycosylation was detected in HCT-116 and HT-29 cells with or without ALG10 knockdown. (**D**) The status of TGFBR2 glycosylation and p-smad2 expression were evaluated in HCT-116 cells with or without ALG10 knockdown or Tunicamycin treatment. (**E**) The nucleus expression of p-smad2 was examined in HCT-116 and HT-29 cells with or without ALG10 knockdown or Tunicamycin treatment. (**F**) The TGFBR1-TGFBR2 and TGFBR1-p-smad2 interaction was measured in HCT-116 and HT-29 cells with or without ALG10 knockdown. (**G**) The mRNA expression of FOXD1, SPHK1, CHST1, and EST2 was determined in HCT-116 and HT-29 cells with or without ALG10 knockdown or Tunicamycin treatment. *n* = 3, ^**^P < 0.01 vs. Control.

Then we explored the underlying mechanisms contributing to *ALG10*-mediated effects on CRC cell stemness. As several lines of evidence have demonstrated the critical role of TGF-β pathway in the maintenance of tumor stemness and ALG3, another ALG family member, has recently been reported to enhance TGFBR2 glycosylation [13], we wondered whether ALG10 held the similar effects on TGFBR2 with ALG3. Firstly, a luciferase assay was conducted in CRC cells with or without *ALG10* knockdown. As shown in Figure 6A and 6B, it was found that *ALG10* knockdown reduced the luciferase activity of luc-smad and luc-TCF-1, indicating that *ALG10* could indeed activate TGF-β signaling in CRC cells. We then evaluated whether ALG10 has an effect on TGFBR2 glycosylation. As expected, we found that ALG10 knockdown significantly downshifted the TGFBR2 bands, but failed to downshift the TGFBR1 bands (Figure 6C). Interestingly, *ALG10* knockdown failed to affect the total level of TGFBR2 (Figure 6C), implying that *ALG10* promotes the activity of TGF-β signaling through glycosylation of TGFBR2 but not its expression. Tunicamycin is widely used as a research tool to block N-linked glycosylation, to verify whether the band shift was caused by a change in the glycans, tunicamycin was used as a positive control and *ALG10* was knocked down in CRC cells. As shown in Figure 6D, the intensity of the lower TGFBR2 bands was decreased, leading to decreased p-smad2 level, this effect was consistent with that of tunicamycin treatment. As nuclear p-smad2 level is responsible for gene transcription, we further investigated the effect of *ALG10* on nuclear transport of p-smad2, and we found the nuclear translocation of p-smad2 was significantly suppressed in both ALG10 knockdown and tunicamycin groups (Figure 6E). Moreover, the interactions between TGFBR1 and TGFBR2, and TGFBR1 and p-smad2, which were the downstream pathway of TGFBR2 underglycosylation, were clarified using Co-IP analysis. As shown in Figure 6F, the interactions between TGFBR1 and TGFBR2, and TGFBR1 and p-smad2 were weakened in colorectal cells with *ALG10* knockdown. In consistent, the expression of downstream effectors (FOXD1, SPHK1, CHST1, EST2) of TGF-β signaling was significantly reduced by both *ALG10* knockdown and tunicamycin treatment (Figure 6G). Thus, our results indicated that downregulation of *ALG10* inhibits TGF-β signaling by disturbing TGFBR2 glycosylation.

### *ALG10* and TGF-β signaling forms a positive *ALG10*/TGF-β regulatory loop by being bound by Smad2

**Figure 7:**
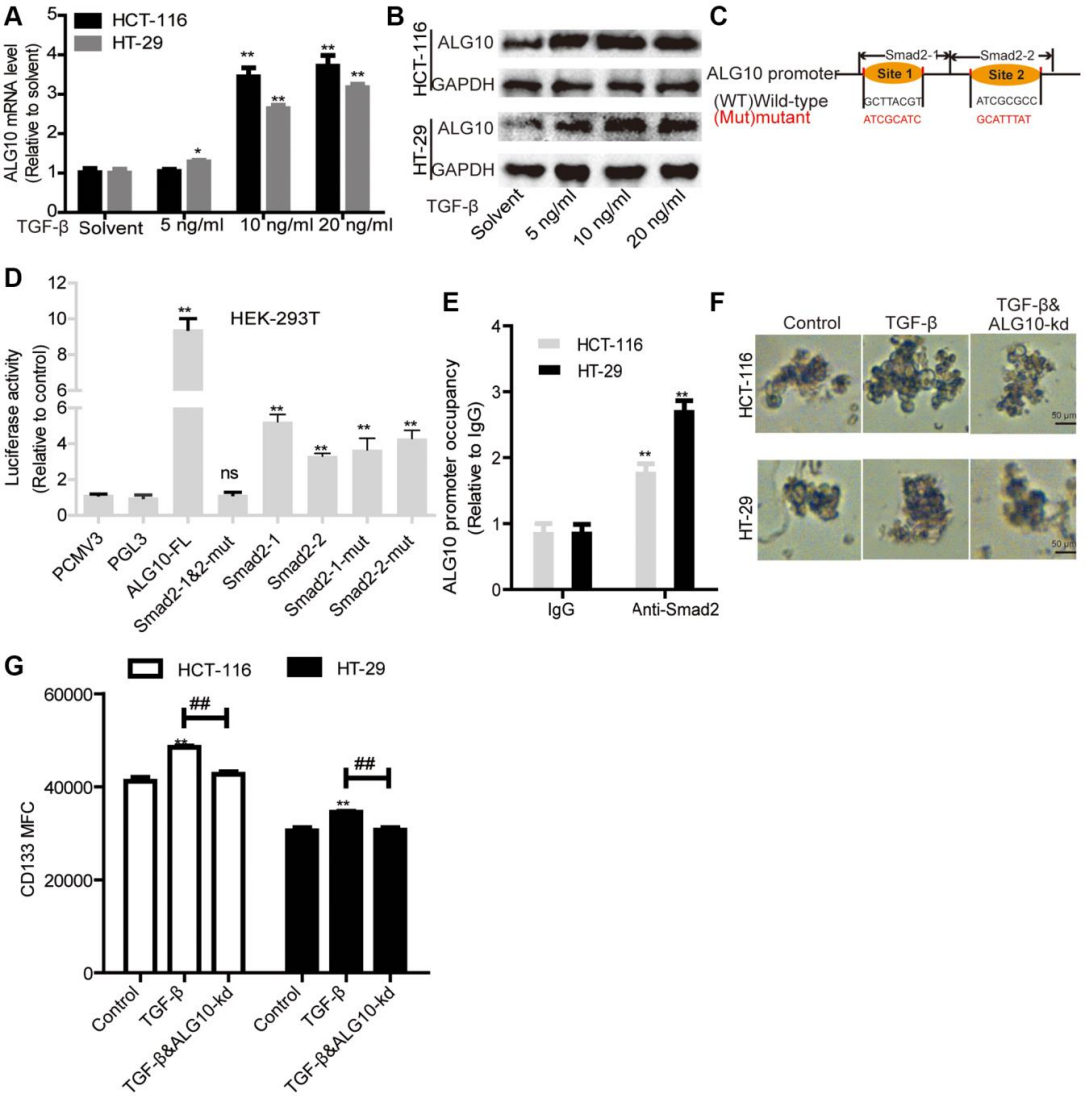
***ALG10* and TGF-β signaling forms a positive *ALG10*/TGF-β regulatory loop by being bound by Smad2.** (**A** and **B**) *ALG10* mRNA and protein levels were examined in CRC cells with the treatment of different concentrations of TGF-β. (**C**) The diagram of Smad2 binding sites on *ALG10* gene promoter. (**D**) Luciferase reporter assay was constructed to detect regulation of Smad2 on the regulation of different truncations of *ALG10* gene promoter as indicated. (**E**) ChIP analysis were performed to determine the binding of Smad2 on *ALG10* gene promoter. (**F**) Sphere size was measured in CRC cells with TGF-β treatment plus ALG10 knockdown or not. (**G**) CD133+ positive cells were evaluated in the cells described in (**F**). *n* = 3, ^**^P < 0.01 vs. Control.

Interestingly, we found that TGF-β could upregulate *ALG10* level in CRC cells (Figure 7A and 7B). Then we explored the underlying mechanisms by which TGF-β upregulates ALG10 level. Bioinformatics analysis (The JASPAR database: http://jaspar.genereg.net/search/?q=&collection=CORE&tax_group=vertebrates) showed that Smad2, the downstream effectors of TGF-β, can bind to *ALG10* gene promoter with two potential binding sites (Figure 7C). Further luciferase reporter assay with or without truncated fragments of *ALG10* gene promoter indicated that Smad2 can bind to both of the two binding sites (Figure 7D), which was further confirmed by ChIP analysis (Figure 7E). Notably, TGF-β could promote the stemness of CRC cells in an ALG10-dependent manner, which was characterized as the change of sphere-formation ability (Figure 7F) and CD133+ subpopulation (Figure 7G).

### TGFBR2 is necessary for *ALG10*-induced stemness of CRC cells

**Figure 8:**
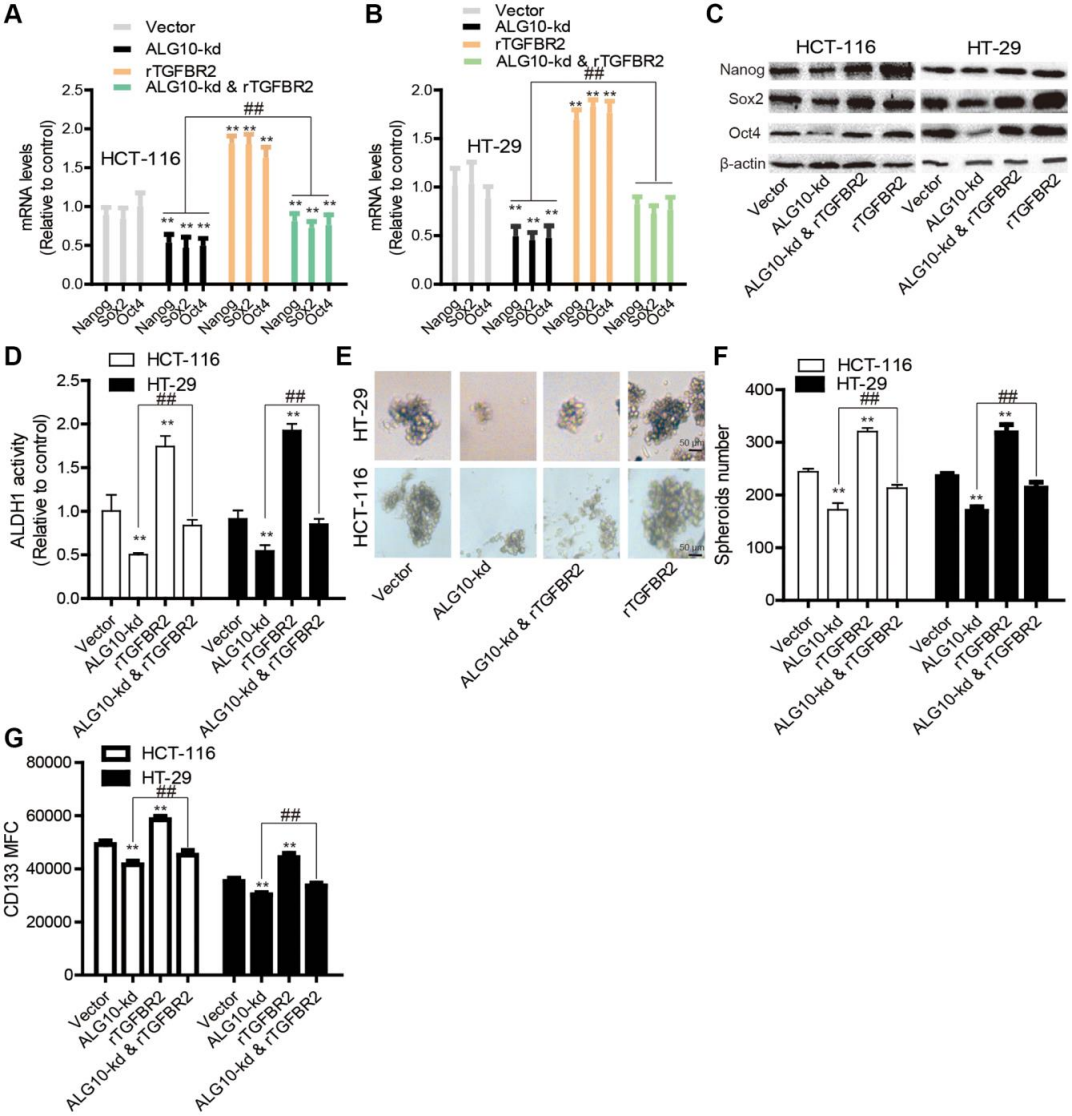
**TGFBR2 is necessary for *ALG10*-induced stemness of CRC cells *in vitro*.** (**A** and **B**) CRC cells with or without ALG10 knockdown were treated with rTGFBR2 or not and then subjected to detect the mRNA levels of stemness markers. (**C**) The protein levels of stemness markers were examined in the CRC cells described in (**A**). (**D**) ALDH activity was measured in the CRC cells depicted in (**A**). (**E** and **F**) The sphere formation ability was evaluated in the CRC cells described in (**A**) by detecting sphere size (**E**) and number (**F**). (**G**) CD133+ cell sub-population was determined in the CRC cells depicted in (**A**). *n* = 3, ^**^P < 0.01 vs. Control, ^##^*P* < 0.01 vs. ALG10-kd & rTGFBR2.

**Figure 9:**
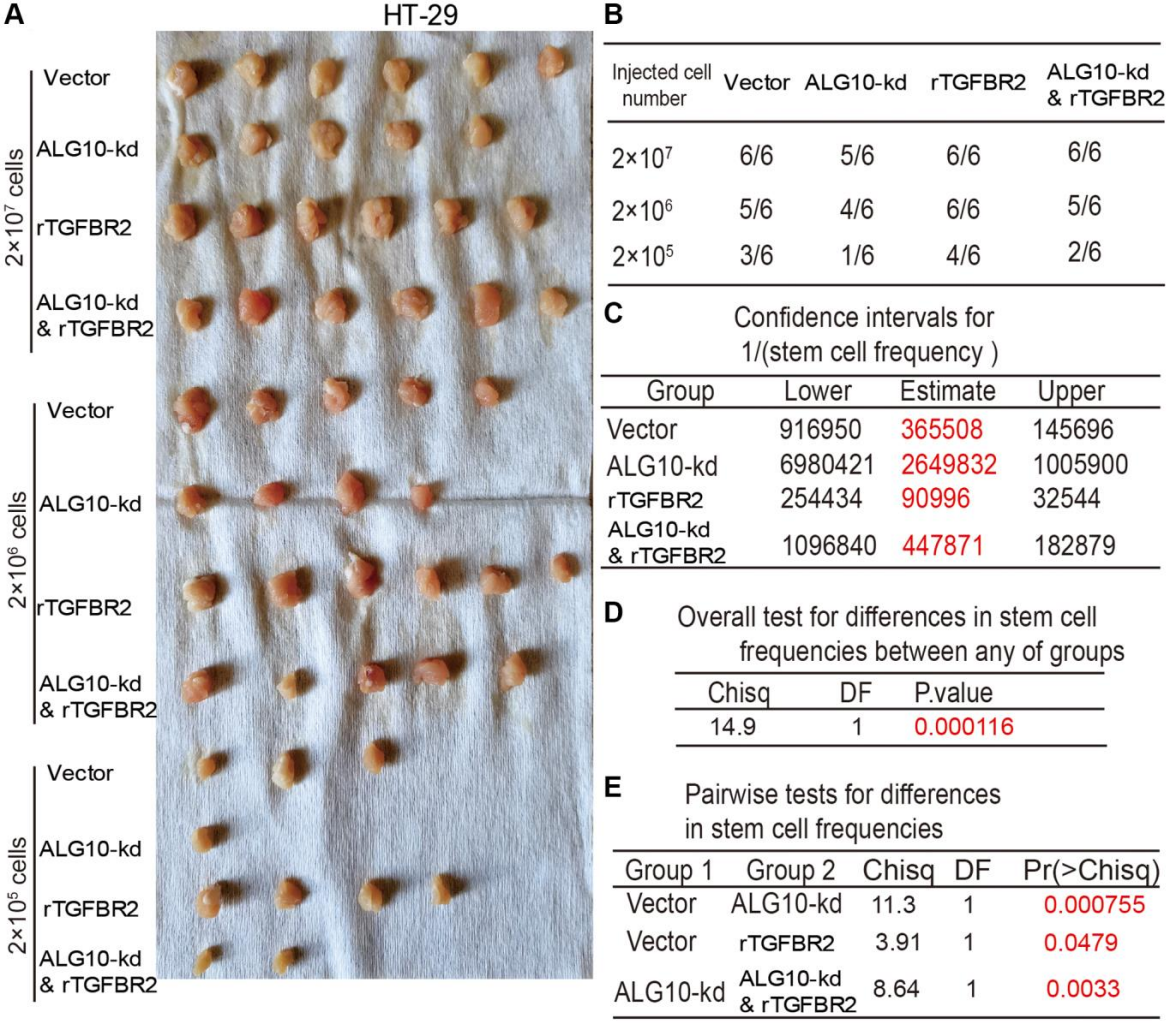
**TGFBR2 is necessary for *ALG10*-induced stemness of CRC cells *in vivo*.** (**A**) Tumor images derived from different types of HT-29 cells as indicated. (**B**) Tumor formation ratio of the HT-29 cells as described in (**A**) was calculated. (**C**) The 1/(stem cell frequency) was determined for the HT-29 as described in (**A**) using an ELDA: Extreme Limiting Dilution Analysis (http://bioinf.wehi.edu.au/software/elda/). (**D**) The difference in stem cell frequencies between any groups described in (**C**) was measured using an ELDA: Extreme Limiting Dilution Analysis (http://bioinf.wehi.edu.au/software/elda/). (**E**) The difference in stem cell frequencies between two groups as indicated was measured using an ELDA: Extreme Limiting Dilution Analysis (http://bioinf.wehi.edu.au/software/elda/).

Finally, we determined whether TGFBR2 was essential for *ALG10*-mediated effects on CRC stemness. Human recombinant protein TGFBR2 was added into cells with *ALG10* knockdown, it was found that addition of TGFBR2 protein partially abrogated *ALG10*-knockdown-mediated downregulation of stemness marker expression (Figure 8A–8C). Additionally, *ALG10* knockdown-induced reduction of both ALDH activity and sphere-formation ability was rescued by TGFBR2 (Figure 8D–8F). Furthermore, the CD133+ sub-population of CRC cells with stemness was attenuated by *ALG10* knockdown through flow cytometry analysis, this effect was attenuated by TGFBR2 (Figure 8G). Notably, TGFBR2 protein enhanced the stemness of CRC cells (Figure 8). In consistent, *ALG10* knockdown-mediated decrease of tumor-initiating ability was rescued by TGFBR2 treatment (Figure 9). Therefore, our results indicate that ALG10 contributes to the stemness of CRC by mediating TGFBR2 glycosylation and thus activating TGFBR2.

## DISCUSSION

The prognosis of CRC patients after radiotherapy and chemotherapy varies significantly, which may be closely related to the development of therapy resistance [14]. Although many studies have been devoted to finding predictive markers of chemoradiotherapy resistance in patients with CRC, no reliable markers have been widely used in clinical practice. Reliable predictors of chemoradiotherapy sensitivity can effectively guide personalized treatment decision-making [15]. At present, glycosylated proteins have become one of the most common tumor biomarkers in clinic [16], such as alpha fetoprotein (AFP) of hepatocellular carcinoma, carcinoembryonic antigen (CEA) of colon cancer and prostate specific antigen of prostate cancer. However, the predictors of glycoprotein related CRC, especially for CRC resistant to radiotherapy and chemotherapy, are still blank.

CSC-like characteristics have been widely proved to be related to chemoradiotherapy resistance and early recurrence of cancer [17]. CSC-like properties promote the biological function of anti-radiotherapy and chemotherapy, which is related to DNA damage repair, hypoxia, and cell cycle arrest [18]. Firstly, as CSC-cells are mostly in the non-dividing G_0_ cell cycle, they are resistant to cell cycle-dependent radiotherapy and chemotherapy, which are more effective for rapidly proliferating cells, especially those in mitotic stage. Secondly, compared with ordinary tumor cells, CSC-like cells hold enhanced DNA damage repairing ability, which is responsible for reducing apoptosis or necrosis induced by DNA damage after radiotherapy and chemotherapy. Finally, hypoxic microenvironment has been proved to play an important role in CSC-induced chemoradiotherapy resistance as oxygen is necessary for the production of cytotoxic reactive oxygen species (ROS), which can damage tumor cells after chemoradiotherapy. Therefore, CSC-like traits are considered to be an important contributor to chemoradiotherapy resistance. In this study, we found that *ALG10* was significantly up-regulated in CRC tissues and associated with a shorter survival in CRC patients. Loss of function experiments showed that downregulation of *ALG10* could suppress the expression levels of several key CSC markers, including Nanog, Sox2, and Oct4, inhibit the ability of sphere formation, and reduce the proportion of CD133+ cell sub-population with stemness. In addition, *in vivo* experiments also confirmed that knockdown of *ALG10* could weaken the tumorigenicity of CRC cells. These findings suggest that *ALG10* acts as a key regulator of CSC-like characteristics in CRC.

CSC-like features can be controlled in various ways. Post-transcriptional modifications, especially glycosylation, have been reported to contribute significantly to CSC-like features in several cancers [19]. Glycosylation is a common post-translational modification of membrane-related and secreted proteins [20]. The special cell surface and extracellular location of glycans are very important for cells to receive signals from the outside, thus glycans play a key role in controlling intercellular communication, signal transduction, and receptor activation. Several key growth factors, such as hepatocyte growth factor (HGF), epithelial growth factor (EGF), vascular endothelial growth factor (VEGF) and TGF-β, play an important role in receptor glycosylation, which further regulates the sensitivity of receptors to ligands, the effectiveness of signal transduction, and cancer progression [21]. Among them, TGF-β pathway is considered to be one of the most important pathways to promote CSC-like traits in different tumor entities, including CRC [22]. There are several important glycosylated proteins in TGF- β pathway, such as TGF- β, TGFBRs and Smads. Previous studies have reported that some glycosyltransferases and glycosidases within TGF-β pathway are tightly related to phosphorylation of TGF-β receptors, such as fucosyltransferase 8 (FUT8) in lung cancer [23] and sialylated N-acetylglucosaminyl-transferase V (MGAT5, 37) in CRC [24]. However, the effect of mannosyltransferase on cellular receipt is unclear. There are three types of N-linked oligosaccharides: Oligomannosidic (high mannose), hybrid, and complex glycan structures [25]. The successful cell surface TGFBR2 transportation requires both complex and oligomannosidic type [21]. ALG family is an important family of mannosyltransferases, for example, ALG3 holds α- 1,3-mannosyltransferase activity and trigger the biosynthesis of lumen oligosaccharide, this is necessary for N-linked glycosylation [26]. And ALG3 distortion leads to the loss of mannose in oligosaccharide chain. Here, we found that *ALG10,* another ALG family gene, is the mostly upregulated gene in ALG family in CRC. However, the biological role of ALG10-induced glycosylation in CRC progression is still unclear. In this study, we found that silencing *ALG10* significantly reduced the glycosylation of TGFBR2. Importantly, the downregulation of *ALG10* will destroy the binding ability of TGFBR2 to TGFBR1, further weaken the phosphorylation of Smad2 and thus inactivate TGF-β signaling inactivation, finally inhibiting the CSC-like characteristics of CRC cells. In addition, the humanized TGFBR2 recombinant protein eliminated the downregulation of CRC stemness induced by *ALG10* knockdown. Overall, our results suggest that ALG10 promotes the CSC-like properties of CRC.

## CONCLUSIONS

In conclusion, our results show that *ALG10* overexpression promotes the glycosylation of TGFBR2 and activates TGF-β signal, thus further promoting the CSC-like traits of CRC. Therefore, the important findings of this study provide new insights into the molecular mechanism of *ALG10* promoting the chemoradiotherapy resistance of CRC cells. Further studies could be constructed to determine the effects of *ALG10* on chemoradiotherapy resistance in CRC, which will facilitate the treatment aiming to ameliorate the chemoradiotherapy resistance of CRC by targeting *ALG10*.

## Supplementary Materials

Supplementary Figure 1
