# Supplementary material for: A novel ALG10/TGF-β positive regulatory loop contributes to the stemness of colorectal cancer
Source: Aging (Albany NY). 2022 Jun 9;14(11):4858–73. doi: 10.18632/aging.204116 (PMC9217715; doi:10.18632/aging.204116)
Supplement: Supplementary Figure 1 [file aging-14-204116-s001.pdf]

SUPPLEMENTARY FIGURE

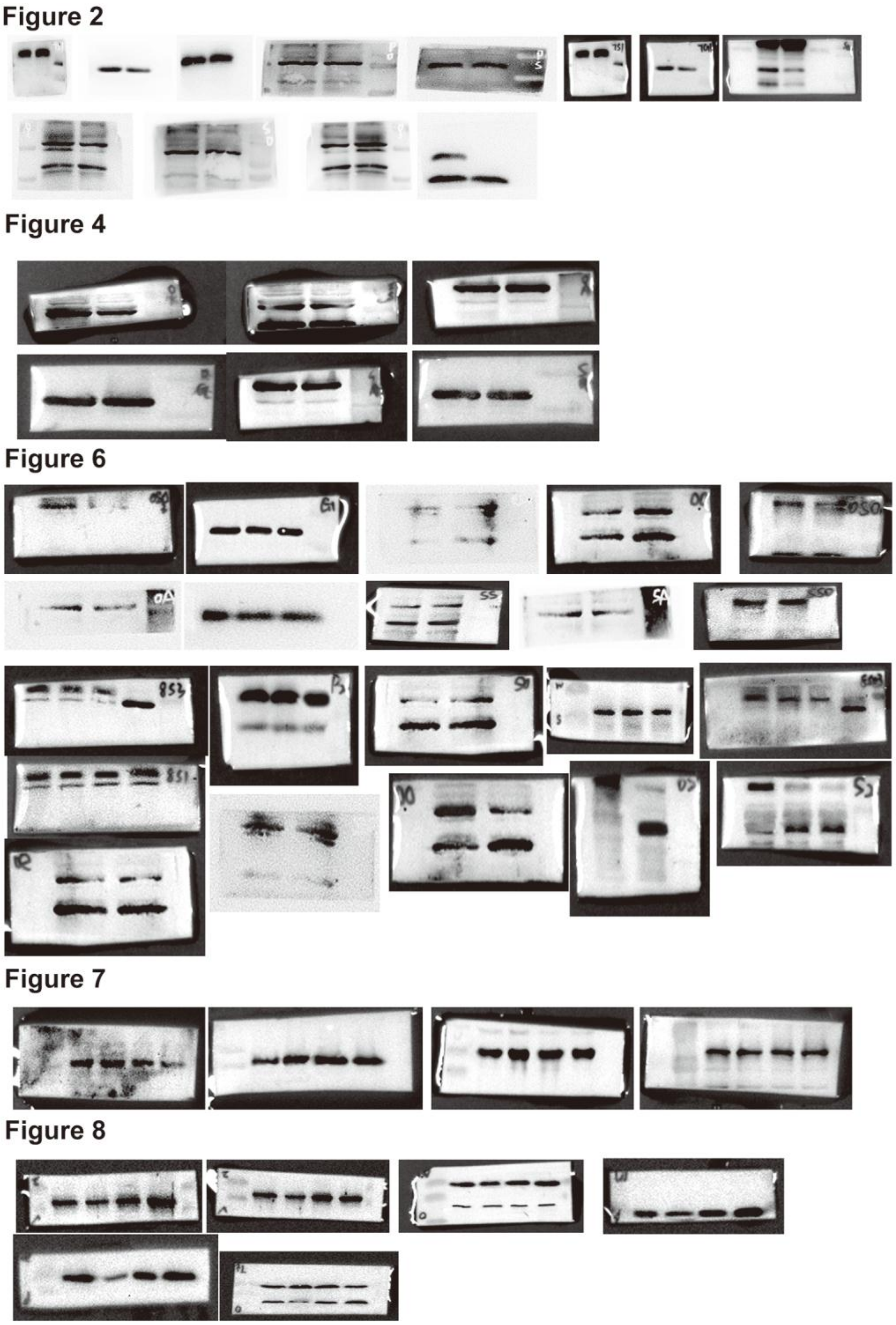

Supplementary Figure 1. The full length uncropped original western blots used in their manuscript.
